# Inflammatory cytokine expression on the ocular surface in the Botulium toxin B induced murine dry eye model

**Published:** 2009-01-30

**Authors:** Lei Zhu, Jikui Shen, Cheng Zhang, Choul Yong Park, Sahar Kohanim, Margaret Yew, John S. Parker, Roy S. Chuck

**Affiliations:** 1 Wilmer Eye Institute, Johns Hopkins University, Baltimore, MD; 2 Henan Eye Institute, Zhengzhou, China; 3 Department of Ophthalmology, Dongguk University, School of Medicine, Ilsan, Korea

## Abstract

**Purpose:**

Inflammation plays an important role in dry eye syndrome. In this study, inflammatory cytokine expression on the ocular surface in the Botulium toxin B (BTX-B) induced mouse dry eye model was investigated.

**Methods:**

CBA/J mice received an injection of saline or 20 milliunits (mU) of BTX-B into the lacrimal gland. Tear production and corneal fluorescein staining were evaluated in all groups before injection and at 3 time points after. The pro-inflammatory cytokines macrophage inhibitory factor (MIF), interleukin-1β (IL-1 β), tumor necrosis factor-α (TNF- α) and interleukin-6 (IL-6) in conjunctival and corneal epithelium were evaluated by real time quantitative PCR and immunohistochemistry.

**Results:**

BTX-B injected mice showed significantly decreased aqueous tear production and increased corneal fluorescein staining at the 1 week and 2 week time points compared with normal control and saline-injected mice. The BTX-B injected mice mRNA expression levels of *TNF-α* and *IL-1β* from conjunctival and corneal epithelial cells increased significantly at two early time points comparing with that of normal and saline injected mice, but *IL-1β* returned to normal levels at the 4 week time point. Saline injected mice showed no difference in mRNA expression of *TNF-α*, *IL-1β*, *MIF*, and *IL-6* on the ocular surface tissue at all time points. Immunohistochemistry confirmed these findings.

**Conclusions:**

BTX-B induced mouse model showed decreased aqueous tear production, increased corneal fluorescein staining, and *TNF-α* and *IL-1β* increased expression on the ocular surface within one month. The patterns seen appeared to mimic those in humans with non-Sjögren’s syndrome keratoconjunctivitis sicca (NS-KCS).

## Introduction

As defined by the International Dry Eye Worskhop, dry eye is a multifactorial disease of the tears and ocular surface that results in symptoms of discomfort, visual disturbance, and tear film instability with potential damage to the ocular surface. It is accompanied by increased osmolarity of the tear film and inflammation of the ocular surface [1]. There is a growing recognition that inflammation plays an important role in dry eye syndrome, which is considered an ocular surface inflammatory disease rather than simply a tear film insufficiency [2]. Increasing levels of proinflammatory cytokines and markers have been observed in both the tear film and ocular surface epithelia of patients with dry eye. Conjunctival biopsy specimens from patients with both Sjögren’s syndrome keratoconjunctivitis sicca (SS-KCS) and non-Sjögren’s syndrome keratoconjunctivitis sicca (NS-KCS) display lymphocytic infiltration and increased immunoreactivity for various markers of inflammation and immune activation, including human leukocyte antigen-DR (HLA-DR), HLA-DQ, intracellular adhesion molecule 1 (ICAM-1), CD40, CD40 ligand and apoptotic marker APO2.7 [3,4]. Proinflammatory cytokines interleukin-1α (IL-1α), and interleukin-1β (IL-1β) have been detected in human conjunctival epithelium and tear film in dry eye [5]. Clinical trials have shown decreased numbers of conjunctival T lymphocytes, HLA-DR, apoptosis markers, and some proinflammatory cytokines such as interleukin 6 in cyclosporine treated dry eye, accompanied by increased tear production and decreased corneal fluorescein staining [6-8]. Increased levels of cytokines have also been observed in ocular surface epithelia in dry eye animal models [9-11]. In an experimental tear-deficiency dry eye mouse model mimicking human non-Sjögren’s syndrome keratoconjunctivitis sicca study, IL-1β, tumor necrosis factor-α (TNF-α), and matrix metalloproteinase-9 (MMP-9) were significantly upregulated in the corneal and conjunctival epithelia [9,10], and both corticosteroid and doxycycline suppressed the expression of MMP-9, IL-1β, IL-1α, and TNF-α [11].

We have previously developed a mouse tear-deficiency dry eye model without lacrimal gland inflammation by injection of Botulium toxin B (BTX-B) into the lacrimal gland [12]. BTX-B injected mice displayed significantly decreased tear production and persistent corneal fluorescein staining with no inflammation of the lacrimal glands. Further, a lacrimal gland inflammatory cytokine gene expression study showed that macrophage migration inhibitory factor (*MIF*) gene expression was elevated in BTX-B injected mice at 2 and 4 weeks time points. Other cytokines such as TNF-α, IL-12, and IL-10 showed no significant change [12]. Lacrimal glands were normal structurally without inflammatory cell infiltration. In this current study, we used the same mouse model to investigate inflammatory cytokine expression directly on the ocular surface.

## Methods

### Animal model

Female CBA/J mice (age 6–8 weeks; Jackson Laboratories, Bar Harbor, ME) were used in accordance with the ARVO Statement for the Use of Animal in Ophthalmic and Vision Research and approved by the Institutional Animal Care and Use Committee of Johns Hopkins University.

Mice were divided into three experimental groups, each group containing eight mice (three for immunohistological analysis and five for cytokine expression analysis). One group was used as a control without any injection into lacrimal glands. The second group of mice was injected with saline into their lacrimal glands and the third group of mice was injected with Botulium toxin B (BTX-B; Myobloc^™^; Elan Pharmaceuticals Inc., South San Francisco, CA) into the lacrimal glands. The mouse model was created in accordance with our previously reported method [13]. In brief, all mice in the second and third groups were anesthetized with Ketamine and Xylazine (45 mg/kg and 4.5 mg/kg, respectively). Saline (0.05 ml) or BTX-B (0.05 ml, 20 mU) was injected into the right lacrimal gland unilaterally through the conjunctiva with custom made 33 gauge needles (Hamilton, Reno, NV) under an operating microscope. All mice were maintained under relatively constant temperature (21 °C  to 24 °C) and humidity conditions (<20%). For the quantitative real-time RT–PCR and immunohistochemistry study, the animals were sacrificed at four time points: before BTX-B injection and at 1, 2, and 4 weeks after injection.

### Measurement of aqueous tear production and corneal fluorescein staining

Measurements of aqueous tear production and corneal fluorescein staining were performed as previously reported [13]. In brief, phenol red-impregnated cotton threads (Zone-Quick; Oasis, Glendora, CA) were applied to the ocular surface in the lateral canthus for 15 s in the unanesthetized mouse, and then the wet threads were measured in millimeters.

Corneal fluorescein staining (1 µl of 1% sodium fluorescein, Sigma-Aldrich, St. Louis, MO) was evaluated under cobalt blue light with a grading system based on area of corneal staining as previously reported [13]. The total area of punctate staining was designated as grade 0 when there was no punctate staining, grade 1 when equal to or less than one eighth was stained, grade 2 when equal to or less than one fourth was stained, grade 3 when equal to or less than one half was stained, and grade 4 when greater than one half or the entire area was stained [14]. All measurements were performed before injection and at 1, 2, and 4 weeks after injection.

### Quantitative Real-Time RT–PCR

Mouse corneal epithelial cells were obtained by scraping and conjunctival epithelial cells were obtained by dissection. Care was taken to dissect the conjunctiva from subconjunctival tissue. Total RNA was isolated from the ocular surface using the RNeasy Kit (Qiagen, Valencia, CA) according to the manufacturer’s instructions. Combining the ocular surface epithelia allowed us to collect sufficient RNA for analysis from each of the 5 animals at each time point. Furthermore, we chose to combine the ocular surface epithelia because an abnormal tear film affects both the corneal and conjunctival surfaces. There were five samples in every group. The concentrations of RNA were determined by UV spectrophotometry. RNA samples were treated with DNase I to exclude genomic DNA contamination. The first-strand cDNA was synthesized from 1 µg of total RNA with oligo d’-T primer using a commercially available kit (SuperScript^TM^ Ш Reverse Transcriptase; Invitrogen, Carlsbad, CA). Samples of cDNA were aliquoted and stored at –80 °C until use.

Real time quantitative PCR was performed and analyzed using the Roche Light-Cycler (Roche Diagnostics Corporation, Indianapolis, IN). Reactions in a 20 µl volume using the SYBR green reaction mix (QIAGEN, Valencia, CA) with 0.5 mmol/l primer. Cyclophilin A was used as a standard for normalization. To quantify the gene copy number, an absolute quantification method was used (Roche). Briefly, PCR was performed using PFU-Taq (Stratagene, Cedar Creek, TX). PCR products were purified and DNA concentrations measured by spectrophotometry. Standard curves for each gene were plotted with quantified cDNA templates during each real-time PCR. Assays were performed in duplicate. The sequences of the PCR primer pairs are listed in Table 1.

**Table 1:** Primer sequences for Real-Time RT PCR.

**Gene**	**GeneBank accession**	**Left primer**	**Right primer**	**PCR Product (bp)**
*IL-1β*	NM_008361	GCCCATCCTCTGTGACTCAT	AGGCCACAGGTATTTTGTCG	229
*TNF-α*	NM_013693	GAACTGGCAGAAGAGGCACT	AGGGTCTGGGCCATAGAACT	201
*MIF*	NM_010798	GTGCCAGAGGGGTTTCTGT	AGGCCACACAGCAGCTTACT	205
*IL-6*	NM_031168	AGTTGCCTTCTTGGGACTGA	CAGAATTGCCATTGCACAAC	190
*Cyclophilin A*	XR_004644	CAGACGCCACTGTCGCTTT	TGTCTTTGGAACTTTGTCGCAA	132

### Immunohistochemistry

For immunofluorescent staining, the cornea and conjunctiva from each group at different time points were harvested by dissection and immersed in 4% paraformaldehyde, fixed overnight at 4 °C, then embedded and frozen in optimal cutting temperature embedding compound (OCT; Tissue-Tek^®^; Sakura, Terrence, CA). Blocks were cryo-sectioned at 10 μm thickness. After drying at room temperature for 15 min, slides were washed twice with phosphate buffered saline (PBS) and incubated with blocking serum for 30 min, then incubated with primary antibodies overnight at 4 °C. The antibodies used were as follows: anti-TNF-α (goat polyclonal IgG; 1:200; Santa Cruz Biotechnology, Inc., Santa Cruz, CA), anti-IL-1β (rabbit polyclonal IgG; 1:400; Santa Cruz Biotechnology, Inc.), and anti-MIF (rabbit polyclonal IgG; 1:100; Santa Cruz Biotechnology, Inc.). Slides were washed with PBS, then secondary antibodies conjugated with either Cy3 or Cy2 (1:100; Jackson Immuno Research, West Grove, PA) were applied and incubated for 1 h at room temperature. After washing with PBS, slides were counterstained with Hoechst 33342 nuclear staining dye (1:4,000; Molecular Probes, Eugene, OR) for 30 s and mounted with Mounting Media (Dako, Carpinteria, CA). Sections were visualized with a fluorescence digital microscope (Eclipse; E1000;Nikon, Tokyo, Japan). Negative control experiments were performed by omitting the primary antibody and incubating in antibody diluent and secondary antibody. Isotype controls were performed by using normal rabbit IgG (1:400) or normal goat IgG (1:100; Santa Cruz Biotechnology, Inc.).

### Statistical analysis

Statistical analyses were performed using SPSS for Windows version 10.0 and the two way *t*-test. Two-way ANOVA was used for the multiple comparisons between different time points. P values of less than 0.05 were considered statistically significant.

## Results

### Aqueous tear production and corneal staining

**Figure 1:**
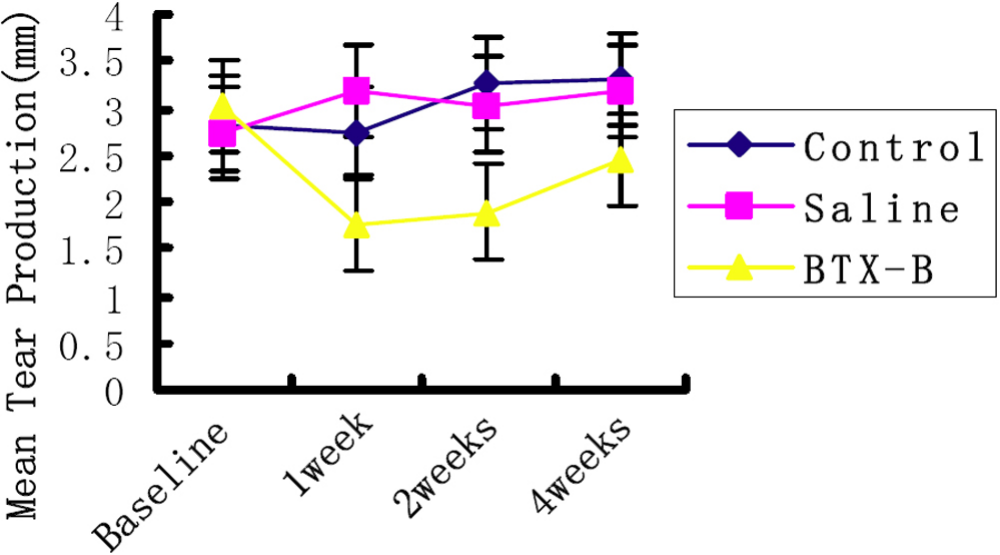
Effect of BTX-B on murine tear production. Mean tear production in all groups. Data represents the mean±SEM in all groups. x-axis: time after injection. The reduction of aqueous tear production in BTX-B injected mice persisted during the observation period, but no change in normal and saline-injected control mice.

All mice retained full blink function after BTX-B injection. BTX-B injected mice showed significantly decreased aqueous tear production at the 1 week (1.77±0.72 mm [mean±SD]) and 2 week (1.89±0.74 mm) time points compared with normal control (2.84±0.88 mm) and saline-injected mice (1 week; 3.13±1.28 mm and 2 weeks; 3.04±1.19 mm post-injection; p<0.01). The reduction of aqueous tear production persisted during the majority of the observation period. Aqueous tear production tended to increase at the 4 week time point (2.45±0.52 mm), but was not statistically significant compared with normal control and saline-injected mice (p=0.097; Figure 1).

**Figure 2:**
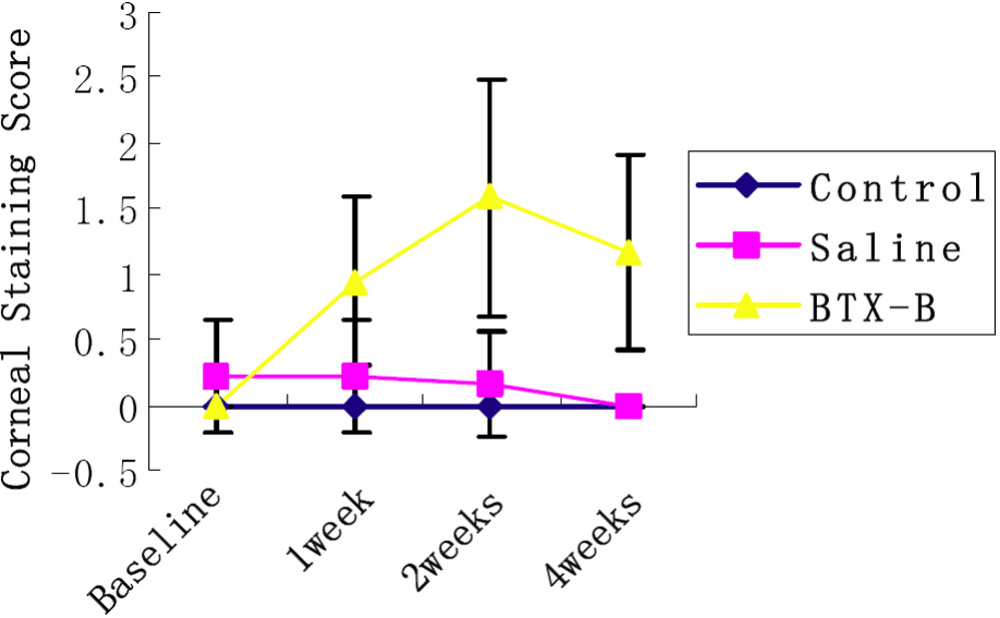
Effect of BTX-B on murine corneal staining. Corneal fluorescein staining scores. Data represents the mean±SEM in all groups. Average score in BTX-B injected mice was significantly increased compared with control and saline-injected mice at all time points (p<0.01). X–axis: time after injection.

**Figure 3:**
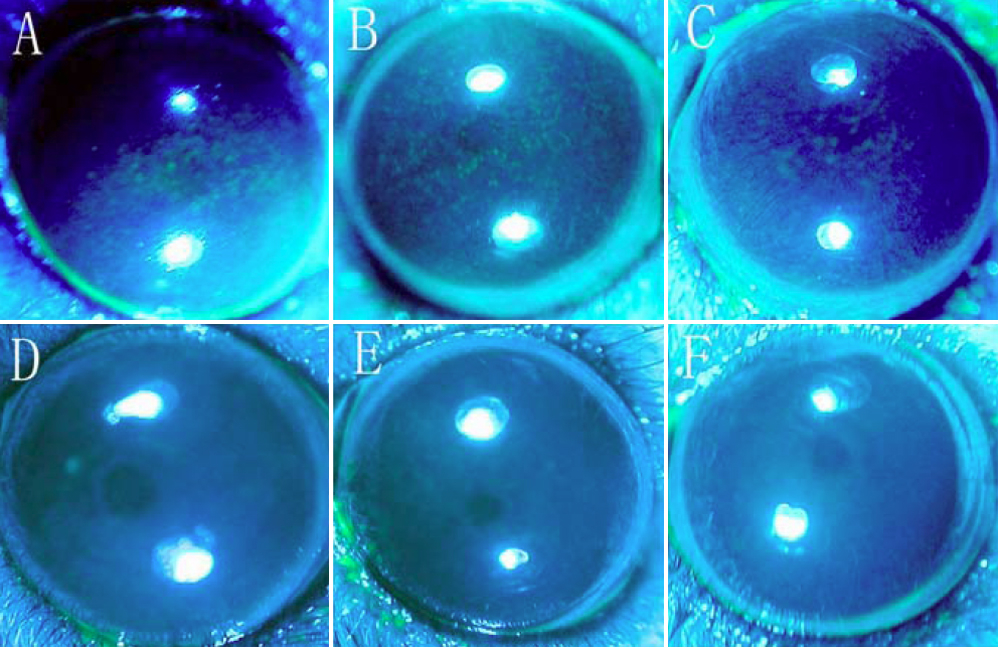
Corneal fluorescein staining in BTX-B injected and normal control mice. **A**-**C**: Corneal staining was seen in BTX-B injected mice 1 week, 2 weeks, and 4 weeks post-injection, respectively. **D**-**F**: No corneal fluorescein staining was noted in normal control mice, saline-injected mice 1 week and 2 weeks post-injection, respectively.

Little or no corneal staining in saline-injected and normal mice was observed at the various time points after injection. In comparison, BTX-B injected mice showed significantly greater amounts of corneal fluorescein staining throughout the observation period (p<0.01; Figure 2 and Figure 3).

### Inflammatory cytokine expression on the ocular surface

**Figure 4:**
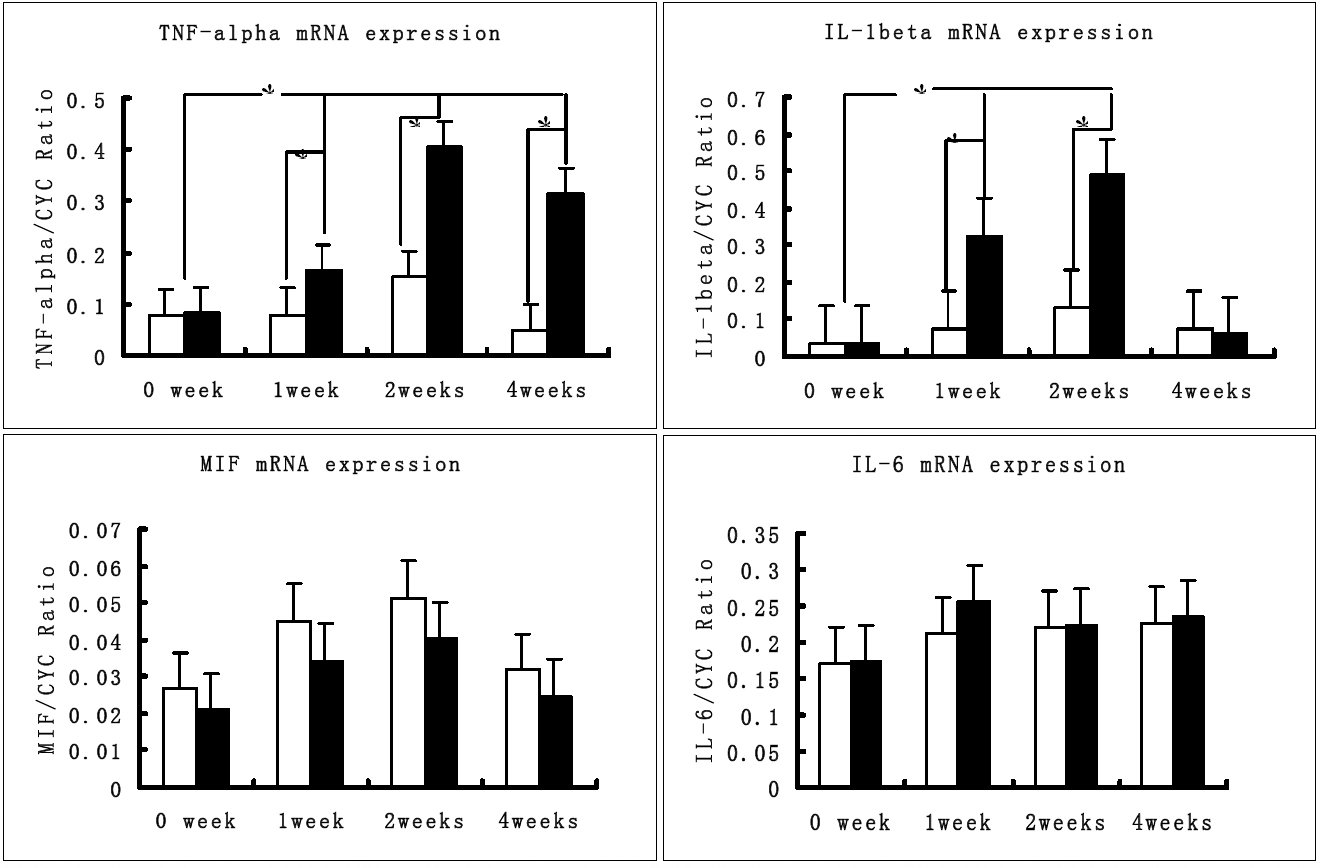
Inflammatory cytokine mRNA expression in ocular surface epithelia of BTX-B injected and normal control mice. mRNA expression levels of *TNF-α* and *IL-1β* in BTX-B injected mice increased significantly at early time points, but normal and saline-injected control mice showed no significant difference. Total RNAs were isolated from cornea and conjunctiva at different time points after BTX-B injection and uninjected contra-lateral eyes were used as controls. Each time point contained five samples. Results are represented as the ratio of cytokine copy number and Cyclophilin A. Error bar: standard deviation of each group. Black bar: BTX-B injected groups, empty bar: Saline-injected groups. The asterisk indicates significant differences between the two groups (p<0.01). The rest without asterisks denote no significant differences between the two groups (p>0.05).

We chose to evaluate mRNA expression of the corneal and conjunctival epithelial cells of the ocular surface. In accordance with our previous results [12], the pro-inflammatory cytokines *MIF*, *IL-1β*, *TNF-α*, and *IL-6* were chosen to evaluate mRNA expression on the ocular surface by quantitative real-time RT PCR at 1, 2, and 4 weeks post-operatively using the housekeeping gene *cyclophilin A* as the internal control. The BTX-B injected mice mRNA expression levels of *TNF-α* and *IL-1β* increased significantly at two early time points comparing with that of normal (0 week) and saline injected mice, but *IL-1β* returned to normal levels at the 4 week time point (p=0.417). Saline injected mice showed no difference in mRNA expression of *TNF-α*, *IL-1β*, *MIF*, and *IL-6* on the ocular surface tissue at all time points (Figure 4).

### Immunohistochemistry

**Figure 5:**
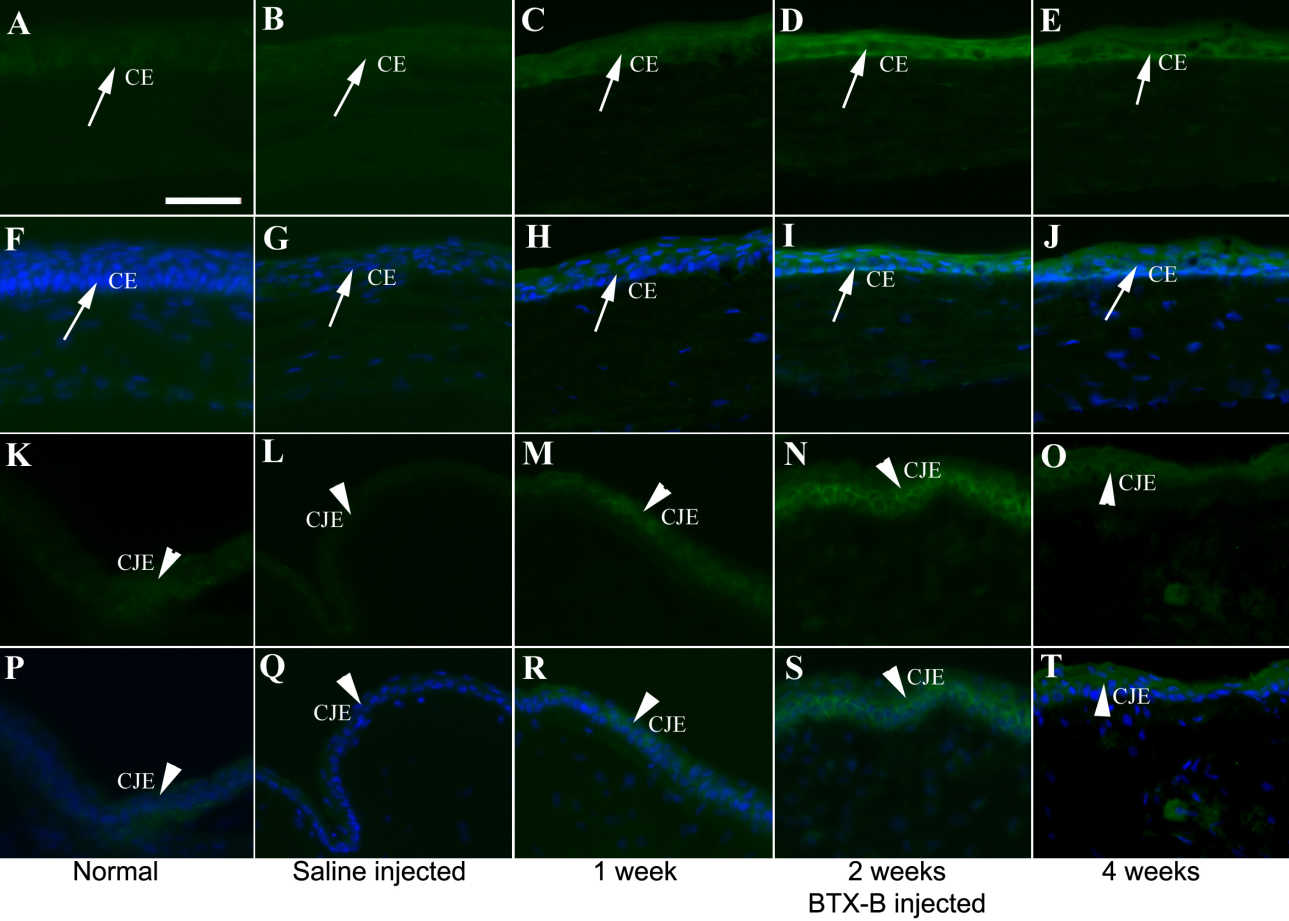
Immunofluorescent staining with TNF-α specific antibody in corneal and conjunctival epithelia. Immunofluorescence staining with TNF- α specific antibody (green) in corneal epithelia (CE: arrows) and conjunctival epithelia (CJE: arrow heads) at all time points. The staining for TNF-α localized in CE and CJE was strong in BTX-B injected mice. Nuclear staining (blue). (**F**-**J**) and (**P**-**T**): merged pictures. Images of isotype and negative control were omitted with no staining. Scale bar: 50 μm.

**Figure 6:**
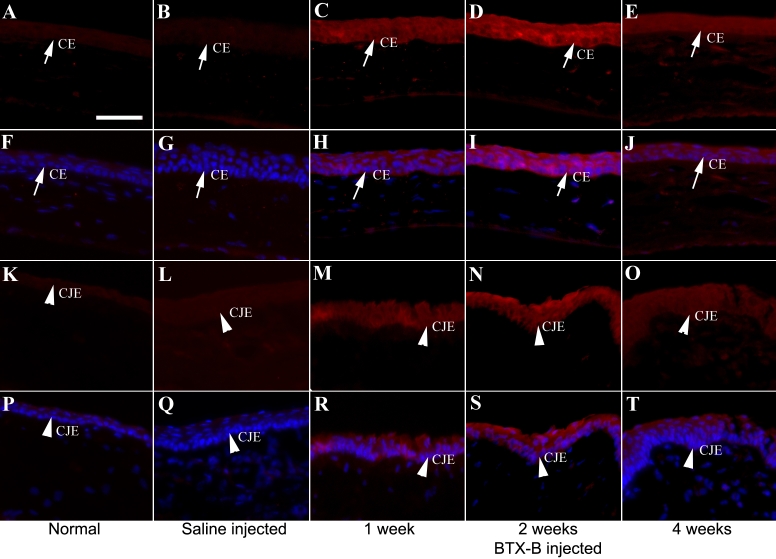
Immunofluorescent staining with IL-1β specific antibody in corneal and conjunctival epithelia. Immunofluorescence staining with IL-1β specific antibody (red) in corneal epithelia (CE: arrows) and conjunctival epithelia (CJE: arrow heads) at all time points. The staining for IL-1β localized in CE and CJE was strong in BTX-B injected mice. Nuclear staining in blue. (**F**-**J**) and (**P**-**T**) merged pictures. Images of isotype and negative control were omitted with no staining. Scale bar: 50 μm.

**Figure 7:**
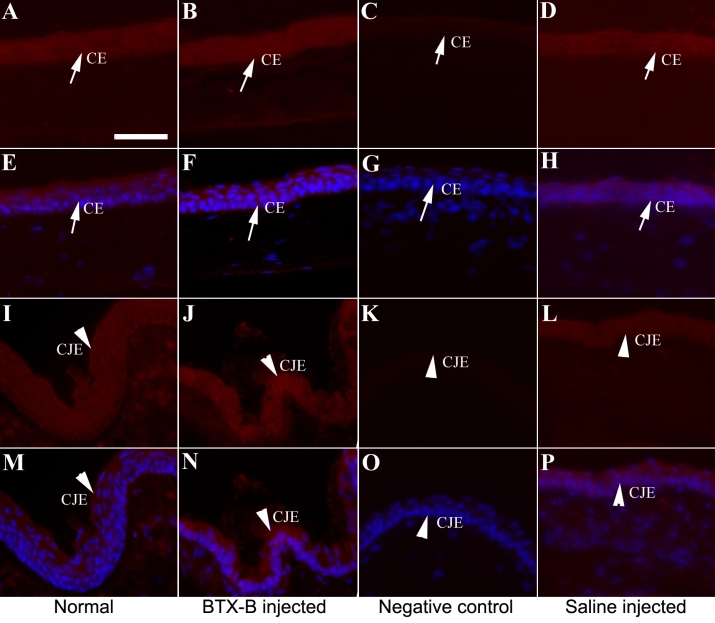
Immunofluorescent staining of MIF in corneal and conjunctival epithelia. Immunofluorescence staining demonstrated the presence of MIF (red) in corneal epithelia (CE: arrows) and conjunctival epithelia (CJE: arrow heads) at all time points. The staining was detected in normal (**A**, **E**, **I**, and **M**) and BTX-B injected mice (**B**, **F**, **J**, and **N**) and saline-injected mice (**D**, **H**, **L**, and **P**) and showed no difference (**C**, **G**, **K**, and **O**). Negative control with no staining. Isotype control was omitted with no staining. Scale bar: 50 μm.

Figures 5, Figure 6, and Figure 7 show typical immunolabeling of normal and saline- injected mouse cornea and conjunctiva with anti-TNF-α, IL-1β, and MIF antibodies. Very weak staining located in corneal and conjunctival epithelia was detected for TNF-α (Figure 5) and IL-1β (Figure 6), while MIF was expressed constitutively (Figure 7) in normal corneas. The corneal (basal, suprabasal, and apical epithelia) and conjunctival epithelia of BTX-B injected mice demonstrated strong staining for TNF-α (Figure 5C-D,H-I,M-N,R-S) and IL-1β (Figure 6C-D,H-I,M-N,R-S) at the 1 and 2 week-time points, whereas the staining decreased at the 4 week time point. MIF expression displayed no change in BTX-B injected mice compared with normal and saline-injected mice.

## Discussion

In this study, we found increased expression of the pro-inflammatory cytokines IL-1β and TNF-α in the corneal and conjunctival epithelium of BTX-B lacrimal gland-injected mice over 4 weeks, coincident with observed tear production decrease and corneal fluorescein staining increase.

We have confirmed that ocular surface inflammation develops in the BTX-B induced dry eye mouse model. Comparing normal and saline-injected mice, *IL-1β* and *TNF-α* mRNA expression in the corneal and conjunctival epithelia significantly increased in BTX-B injected mice at 1 and 2 weeks after injection, and decreased back toward baseline at 4 weeks, especially *IL-1β*. Immunohistological staining results support the findings of the gene expression study. These changes were also consistent with observed alterations in tear production.

In a previous study, we found that IL-1β and TNF-α expression in the lacrimal gland did not increase, but they were increased in corneal and conjunctival epithelia in BTX-B injected mice in the present study. The role of these cytokine increases remains unclear. One possible explanation for the disparity in inflammatory cytokine expression between the lacrimal gland and the ocular surface is that BTX-B imparts a pure aqueous deficiency, and the inflammatory changes on the ocular surface are secondary reactive changes. Previous work supports this hypothesis in that secondary tear hyperosmolarity and micro-abrasive effects of blinking may lead to upregulation of cytokines such as TNF-α and IL-1 in the ocular surface. Further studies propose that one of the original stimuli may come from the tear deficiency-related hyperosmotic stress on the ocular surface, which activates stress JNK, ERK, or P38 MAPK signaling pathways, and the activated MAPK signaling pathway plays a role in the induction of inflammatory cytokines IL-1β and TNF-α [9,15].

An interesting finding in this study is that MIF was expressed constitutively in corneal and conjunctival epithelia of our mice, and that there was no significant change in the BTX-B-induced dry eye mouse model. In our previous study, we found that MIF was elevated in the lacrimal gland and tear fluid at 2 weeks after BTX-B injection [12]. MIF is a potent activator of T-lymphocytes in some other inflammatory diseases [16,17], but the function of MIF in the lacrimal functional unit is remains unclear. However, it has recently been demonstrated that MIF, abundantly expressed in neovascularized mouse corneas, has an angiogenic role in inflammatory corneal neovascularization. Furthermore, homozygous MIF-deficient mice displayed significantly less corneal neovascularization and stromal inflammatory cell infiltration [18]. Further studies are underway incorporating both overexpressing MIF transgenic as well as MIF knockout mice in hopes of better understanding the role of this potent inflammatory mediator in our dry eye mouse model.

In conclusion, we have demonstrated the increased expression of the pro-inflammatory cytokines IL-1β and TNF-α in the corneal and conjunctival epithelium of lacrimal gland in BTX-B-injected mice over 4 weeks, as well as decreased tear production and increased corneal fluorescein staining.
